# Leveraging Social Media to Predict COVID-19–Induced Disruptions to Mental Well-Being Among University Students: Modeling Study

**DOI:** 10.2196/52316

**Published:** 2024-06-25

**Authors:** Vedant Das Swain, Jingjing Ye, Siva Karthik Ramesh, Abhirup Mondal, Gregory D Abowd, Munmun De Choudhury

**Affiliations:** 1 Khoury College of Computer Sciences Northeastern University Boston, MA United States; 2 College of Computing Georgia Institute of Technology Atlanta, GA United States; 3 College of Engineering Northeastern University Boston, MA United States

**Keywords:** social media, mental health, linguistic markers, digital phenotyping, COVID-19, disaster well-being, well-being, machine learning, temporal trends, disruption

## Abstract

**Background:**

Large-scale crisis events such as COVID-19 often have secondary impacts on individuals’ mental well-being. University students are particularly vulnerable to such impacts. Traditional survey-based methods to identify those in need of support do not scale over large populations and they do not provide timely insights. We pursue an alternative approach through social media data and machine learning. Our models aim to complement surveys and provide early, precise, and objective predictions of students disrupted by COVID-19.

**Objective:**

This study aims to demonstrate the feasibility of language on private social media as an indicator of crisis-induced disruption to mental well-being.

**Methods:**

We modeled 4124 Facebook posts provided by 43 undergraduate students, spanning over 2 years. We extracted temporal trends in the psycholinguistic attributes of their posts and comments. These trends were used as features to predict how COVID-19 disrupted their mental well-being.

**Results:**

The social media–enabled model had an F1-score of 0.79, which was a 39% improvement over a model trained on the self-reported mental state of the participant. The features we used showed promise in predicting other mental states such as anxiety, depression, social, isolation, and suicidal behavior (F1-scores varied between 0.85 and 0.93). We also found that selecting the windows of time 7 months after the COVID-19–induced lockdown presented better results, therefore, paving the way for data minimization.

**Conclusions:**

We predicted COVID-19–induced disruptions to mental well-being by developing a machine learning model that leveraged language on private social media. The language in these posts described psycholinguistic trends in students’ online behavior. These longitudinal trends helped predict mental well-being disruption better than models trained on correlated mental health questionnaires. Our work inspires further research into the potential applications of early, precise, and automatic warnings for individuals concerned about their mental health in times of crisis.

## Introduction

The effects of global-scale crises on individual well-being often reach far beyond the actual incident. The onset of the COVID-19 pandemic highlighted this phenomenon. Within the first 7 months of COVID-19, we witnessed increased stress, anxiety, and depression across the world [1]. These outcomes can be explained by direct effects of the infection, for example, hospitalization [2], and indirect effects, for example, loss of family [3], anticipation of infection [4], or job loss [5]. Even the response to crises can exacerbate risks to well-being. One of the key interventions against the spread of COVID-19 was social isolation, but this recommendation also led to worsening mental health by increasing loneliness [6]. In addition, these disturbances can deteriorate an individual’s physical health by hindering his or her activity [7]. Not only do such disruptions worsen a person’s well-being but they may also discourage him or her to comply with crisis management guidelines, thereby worsening the crisis itself. During the severe acute respiratory syndrome epidemic, the anticipated distress of quarantining led to individuals deviating from recommended public health behaviors [8]. Infectious diseases are not the only kind of disasters that trigger depleted mental well-being. This phenomenon has been documented in other kinds of natural and man-made disasters [9-11]. Therefore, to preserve public well-being holistically, we need to enable ways to extend support and coping mechanisms to affected individuals during crises.

To prepare support for disaster well-being, it is important to identify the people whose lives were impacted as a consequence. Typically, this has been done through national-level reviews and surveys. However, meta-analyses have several inferential limitations [12], while surveys tend to have limited or biased coverage [13]. In commentary on mental health consequences after COVID-19 by Tušl et al [14], they posited that social media provides a unique opportunity to “identify and mitigate the immediate and long-term adverse psychological consequences associated with global crises.” People provide self-initiated perspectives on social media that can deepen our understanding of mental well-being states longitudinally [15]. Our paper demonstrates the efficacy of modeling individually contributed social media data to predict COVID-19–induced mental well-being disruptions.

The COVID-19 pandemic occurred at a time when activities on social media were already mainstream and so were the analyses of these activities. It represents an exemplary crisis scenario where we can leverage big data to gain a better understanding of the health impacts. Since the pandemic, we have already seen the use of social media analyses to explain population-level effects on mental well-being. Research on public social media, such as Twitter, has found an increase in psycholinguistic expressions of depression, anxiety, and stress pre- and post onset of COVID-19 [16,17]. Although public social media gives a strong signal of population-level sentiment, it is marred by self-presentation biases of the users posting the content, which can in turn misrepresent actual mental states [18,19]. Our research explores an alternative and complementary approach to provide individualized care by modeling longitudinal linguistic trends on users’ private social media contributed voluntarily. Private web-based personas (eg, on Facebook) have been noted to be less influenced by people’s impression management goals, and thus are likely to represent their actual selves, as opposed to idealized versions [20-22]. We specifically scoped our study to undergraduate students in the United States because these people have disproportionately suffered from worsening mental well-being challenges [23] and were more vulnerable to distress during COVID-19 [24,25]. We developed our machine learning model based on 4124 Facebook posts provided by 43 undergraduate students, spanning August 2019 through November 2021. We modeled and validated these data on an extensive ground truth battery that captured a variety of mental health states. We predicted self-reported mental well-being disruption with an *F*_1_-score of 0.79, which was a 34% improvement over predictions using correlated mental health questionnaires. In addition, to minimize data usage, we also discuss the varying data lengths required to make acceptable predictions. Through this study, we motivate future paradigms where concerned individuals can contribute private social media data to gain early insight into their risk of crisis-induced disruptions to mental health.

## Methods

### Recruitment

Historically, university students have been vulnerable to different mental health concerns such as anxiety, depression, and suicidality [23]. Crises such as COVID-19 have been shown to exacerbate mental health challenges among this group [25]. We recruited undergraduate students from a large urban university in the United States. All participants enrolled as students before the COVID-19–induced campus closure (March 15, 2020) and were students up until the time of recruitment. The recruitment was conducted between September and October of 2021. On joining, the participants were directed to our study portal hosted on the campus network. Participants signed in securely through their student credentials and were provided with a unique study identifier (eg, P112). The portal guided participants to complete an onboarding questionnaire and follow instructions to share their data. We provided participants with different mental health resources to access in case they were in need. Initially, we found that 87 students were interested to participate in the study. To minimize the sparsity of social media data, we retained only those students who had on average at least 1 post per month or 1 active event (such as reacting to someone else’s post). Finally, 43 students participated in the study.

### Ethical Considerations

The study was approved by the Georgia Institute of Technology institutional review board (Protocol #H21051). The consent form was distributed online and potential participants were informed about the nature of data we required for the analysis and the type of analysis we would conduct. The participants were also informed of their ability to delete, or withhold, any part of their data they felt uncomfortable sharing, such as specific periods or types of data (eg, comments). All the data were deidentified using a unique study identifier and mapping to these IDs was kept on a separate server from the one that stored the data. We remunerated students with US $35 for participating in our study and providing their data.

### Data

Next, we describe the various types of data collected through the above recruitment and how they were processed for the ensuing machine learning modeling.

#### Survey

As a part of the data collection effort, the participants completed an extensive battery of survey questionnaires related to COVID-19 and general mental health. We used the Coronavirus Health and Impact Survey (CRISIS) [26] questionnaire to ask the participants how their health was before and after the COVID-19 pandemic. This instrument included 2 separate items to inquire about the participants’ mental health before and after COVID-19. The first item asked participants to rate their “overall Mental/Emotional health before the COVID-19 pandemic,” and the second item asked for them to rate the same but in a more recent frame, “overall Mental/Emotional health lately.” For our study, if the change in mental health scores reduced since their pre–COVID-19 ratings, they were assigned the *disrupted* label. Our machine learning (ML) model was designed to predict whether a user is in the disrupted class using his or her social media data. To validate our model, we used other instruments to measure participants’ psychosocial characteristics. Particularly, we used Patient Health Questionnaire-9 to measure *depression* [27], Suicidal Behaviors Questionnaire—Revised to measure *suicidal behavior* [2], Brief Resilience Scale to measure *resilience* [28]*,* and Patient-Reported Outcomes Measurement Information System to measure *anxiety* and *social isolation* [29]. Table 1 summarizes the different mental health constructs measured as a part of our data collection.

**Table 1:** Summary of the survey instruments used, their purpose in our study, and the class distribution for our machine learning models.

Survey instrument	Class split	
	Concerned	Unconcerned
CRISIS^a^—COVID Disruption^b^	Stable: 23	Disrupted: 20
PROMIS^c^—Anxiety^d^	Low risk: 23	Medium-high: 20
PROMIS—Social Isolation^d^	Low risk: 22	Medium-high: 21
PHQ-9^e^—Depression^d^	No risk: 14	Mild-severe: 29
SBQ-R^f^—Suicidal Behavior^d^	Negative screen: 30	Positive screen: 13
BRS^g^—Resilience^h^	—^i^	—

^a^CRISIS: Coronavirus Health and Impact Survey.

^b^Target label for machine learning.

^c^PROMIS: Patient-Reported Outcomes Measurement Information System.

^d^Comparison: score used to model target as a baseline. Validation: class label used to benchmark features for machine learning.

^e^PHQ-9: Patient Health Questionnaire-9.

^f^SBQ-R: Suicidal Behaviors Questionnaire—Revised.

^g^BRS: Brief Resilience Scale.

^h^Comparison.

^i^Not applicable.

#### Social Media

**Figure 1:**
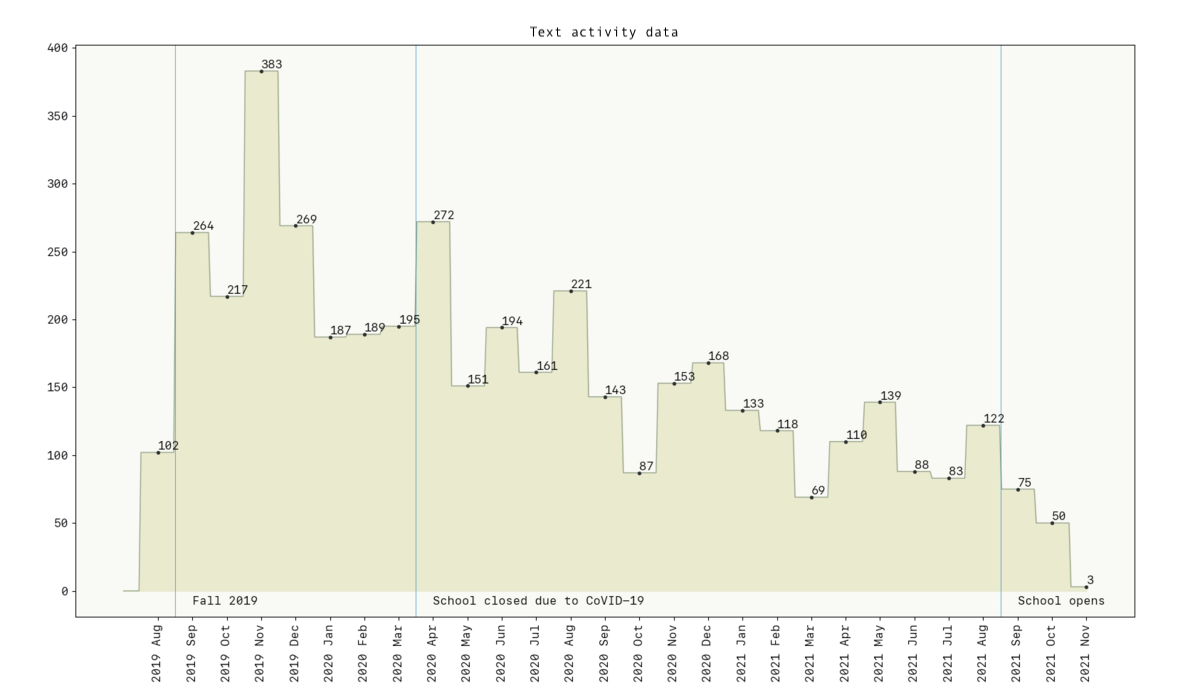
Gradual decline in Facebook content generated by our participants over the study period. Apart from this trend, we did not witness any notable interruption due to COVID-19 in terms of total posts and comments.

After completing the survey, the participants were asked to visit their Facebook profile and download a subset of their information—posts, comments, and reactions. We neither requested nor stored any instant messages or media data (eg, images and videos). Then they submitted these data on our study portal. They were instructed to provide data only since August of 2019—1 semester before the events of COVID-19. The portal deidentified the participants’ name with their participant identifier. In addition, any other labeled name entities (such as the receiver of the comment) were also deidentified. The portal showed participants a sample of the information we stored for further analysis. These data were stored in a secure server hosted at the lead authors’ institute and for accessing these sensitive data, we followed the Principle of Least Privilege. We observed some gradual decline in posts and comments over the period, but we did not see any aberration due to the crisis itself (Figure 1). On the other hand, engagement on Facebook remained relatively stable before and after the school closure. In totality, our data set included 4124 text-based posts (including status updates and comments).

### Machine Learning

#### Feature Engineering

We built a primary feature set with the linguistic inquiry and word count (LIWC) lexicon, a validated dictionary of word categories that has been used extensively in prior research on social media [30]. This approach is inspired by other studies that have used both public and private social media data to predict mental states [17,31]. We referred to the selection of 50 LIWC categories by De Choudhury et al [32] to describe each post in our data set in terms of the occurrence of these 50 psycholinguistic attributes. To extract longitudinal trends in people’s behaviors and attitudes, we sought to assign psycholinguistic attributes temporally. College students use social media quite often, but not all usage involves posting content. Even though our data spanned over 2 years, a large number of days did not have any posts. Alternatively, on some days, users might even have multiple posts. About 51 days had the same user posting multiple times. On these days, we averaged the score for each feature. To account for days without posts, we used a 4-week rolling window that averaged feature values within that window. The window rolled by 1 day. The application of this rolling window technique assumes that people’s mental states gradually peak and trough and social media posts represent only an instance but not the people’s feelings across periods [31]. In this way, we obtained a time series for every user for each LIWC feature. Then, we extracted time series features such as *absolute energy*, *kurtosis, longest strike above mean, number peaks, last location of maximum,* and *autocorrelation*. We computed these features using the TSFRESH package for Python [33]. These time series–based features were our final set.

#### Model Parameters and Evaluation

Since we had more than 2 years of social media data, we decided to identify the optimal window of the above time series data to maximize the performance of our classifier. For this, we considered 2 parameters, the *latest month* (posts after this period were ignored) and *number of months* available (posts before this period were ignored). We built various models to predict mental health disruption by varying these parameters. We effectively performed a grid search varying these 2 parameters to find the best-performing combination. This document reports the best-performing models. All social media models used gradient boost [34] classifiers with 100 estimators, maximum depth of 10, and learning rate of 0.1. To evaluate these models, we used leave-one-participant-out validation—a decision stemming from the relatively smaller number of participants in our data set. As a result, our evaluation considered each participant as a test case while the classifier was trained on data from all other participants.

#### Baseline Model

A reasonable approach to anticipate mental health disruptions would be through survey to identify an individual’s mental state. People with anxiety and depression could find it difficult to cope with the external distress caused by a crisis event [25,35]. A community may consider using validated surveys to screen for these mental states [2,18,27]. However, it can be challenging to scale these surveys over large populations, such as an undergraduate campus. It can also be challenging to receive frequent responses to such surveys. Therefore, we compared the efficacy of our social media–enabled model against the self-reported baseline model. Our participants completed these surveys on recruitment. We used participant mental health scores for *resilience, depression, anxiety, social isolation, and suicidal behavior* to develop a baseline prediction model. We used gradient boost [35] along with a leave-one-participant-out validation method to test this model.

#### Validation Model

To provide external validity for our choice of features (derived from social media), we first tested the explanatory power of these features by building models to classify depression, anxiety, social isolation, and suicidal behavior. Prior research provides sufficient evidence of acceptable ML performance for similar constructs [36,37]. In our results, we report the performance of these models to validate that our features are meaningful to train ML models for predicting the mental well-being status of university students.

## Results

The key results from our study are summarized in Table 2.

**Table 2:** Summary of the prediction performance of different machine learning models^a^.

Features and target				Precision		Recall		*F*_1_-score
**Baseline**								
	**Mental health scores from self-reported surveys**							
		MH^b^ disruption	0.60		0.59		0.59	
**Social media**								
	**Temporal trends in psycholinguistic aspects of speech on social media**							
		MH disruption	0.76		0.76		0.76	
**Validation**								
	**Temporal trends in psycholinguistic aspects of speech on social media**							
		Anxiety	0.92		0.92		0.92	
		Depression	0.84		0.85		0.85	
		Social isolation	0.88		0.88		0.89	
		Suicidal behavior	0.92		0.92		0.93	

^a^Predictions with social media data performed better than the baseline to predict disruption to mental health, and we found it highly effective in predicting other psychosocial constructs.

^b^MH: mental health.

### Social Media–Enabled ML Model

**Figure 2:**
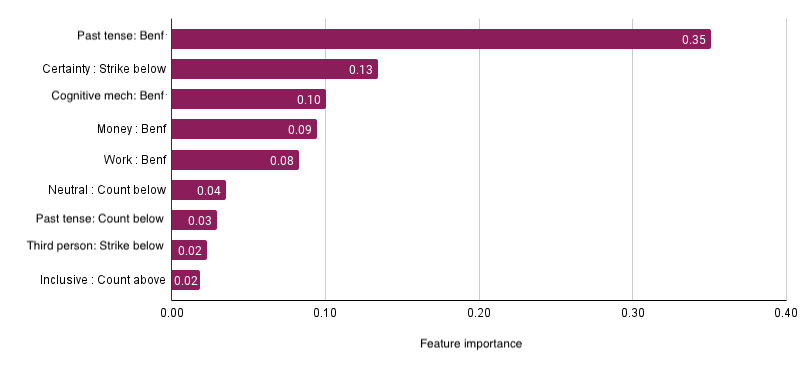
The top 10 features contributing to the best social media–enabled model to predict mental health disruptions. These features represent temporal trends of specific psycholinguistic attributes in the language of participants. The bars represent their feature importance.

We first built baseline models using scores in self-reported survey instruments (anxiety, depression, social isolation, suicidal behavior, and resilience) to predict mental health disruptions. To compute the performance, we pooled all the predictions (1 per person) and compared it against the ground truth. We found that these predictions performed slightly better than chance (*F*_1_-score=0.59). In comparison, features extracted from social media were much better at predicting disruptions (*F*_1_-score=0.76). As described in Table 2, we found that social media had a high positive predictive value (recall=0.76) and high true-positive rate (recall=0.76). Our results show that archival social media data can be provided by students to monitor their mental health during crises in a dynamic and scalable way. The 5 most important psycholinguistic trends were *past tense* (*Benford correlation*), *certainty* (*continuous strike below mean*), *cognitive mechanism* (*Benford correlation*), *money* (*Benford correlation*), and *work* (*Benford correlation*). Note that the prominent temporal trend in these models was the *Benford correlation*, which is known to identify anomalies in real data [38]. Among important linguistic attributes, *past tense* can indicate ruminating [18]; *certainty, money, and work* can all indicate changes in anxiety [39]. Figure 2 shows the top 10 features along with their contribution to the model.

### Validity of Temporal Trends in Psycholinguistic Aspects of Speech

To ensure that our features are meaningful representations of users’ mental health, we developed additional models that predicted different mental health states.

Overall, we found commendable results in predicting students at risk of concerning mental health: anxiety (*F*_1_-score=0.92), depression (*F*_1_-score=0.98), social isolation (*F*_1_-score=0.89), and suicidal behavior (*F*_1_-score=0.93). Refer to Table 2 to see the performance of our validation models across other metrics. These results are comparable with results from other studies predicting mental health status with similar data sources. Saha et al [37] predicted mood instability of 23 college students with a maximum *F*_1_-score of 0.83. Islam et al [36] predicted depression markers in Facebook posts with a maximum *F*_1_-score of 0.73. Thus, extracting temporal psycholinguistic trends from personal social media can be a versatile source to approximate mental health states of individuals.

### Post Hoc: Grid Search Analysis

**Figure 3:**
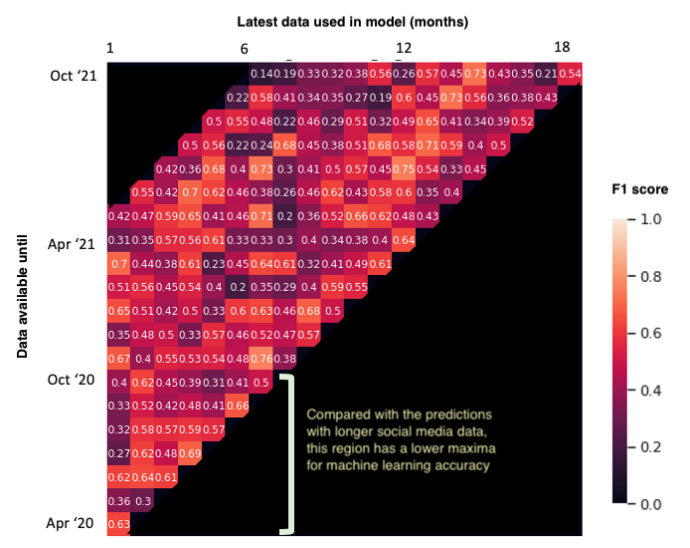
The x-axis shows the latest month used in feature extraction and the y-axis shows the total number of months included preceding the latest month.

In total, we had approximately 19 months of social media data since the campus went on lockdown because of the COVID-19 crisis. Participants reported their changes in well-being by reflecting over this entire period. As described in the Methods section, we searched through the entire time series and used variable lengths of data to understand when the signals for disruption emerge. Specifically, we were varying 2 parameters, *latest month* (April 2020 to October 2021) and *number of months* available (1-18 months). Figure 3 shows the *F*_1_-score for every combination of these 2 parameters. We ignored periods where we did not have sufficient data—particularly, when latest month was closer to October 2021 and number of months available were fewer. The sparsity of posts in this period could be due to the natural decline in Facebook posts over time (Figure 1). Collectively, we trained 128 different versions of the ML model. We analyzed the findings from this grid search using an ordinary least squares regression to inspect whether the 2 parameters had any association with the *F*_1_-score. We found that neither *latest month* (*P*=.31) nor *number of months* (*P*=.62) had any relationship with the *F*_1_-score. Visually analyzing Figure 3 confirms this as increasing values on the x-axis do not indicate more accuracy. At the same time, the peak accuracy during earlier months is relatively lower. We observed this through the lack of bright spots in between April 2020 and October 2020. In comparison, after these 7 months, the data appear to have enough diversity for our ML model to identify the most predictive trends in the data.

## Discussion

### Principal Findings

Large-scale crises can immediately lead to devastating consequences. In addition to the physical risk posed by a crisis event, individuals are likely to experience disruptions to their mental health as they try to cope with the disaster [1,6-11]. A key step in preparing for disaster well-being is to identify people whose mental well-being may have been impacted. Modeling activity on public social media already presents promise for building automatic models to infer trends in population well-being during times of crisis such as COVID-19 [16,17]. However, these applications are likely to ignore individual needs. Our research extends this body of work by demonstrating the feasibility of private social media, such as Facebook, as a potential indicator of disrupted well-being during crisis. We have shown that the psycholinguistic markers of the language used in personal social media can present temporal trends, which in turn help predict disruptions to mental well-being.

Through our study, we highlight the potential of personal social media to support disaster well-being. Additional training of ML models can help design applications where individuals can voluntarily choose to have their status dynamically monitored by providing a period of their social media data. As a result, health care providers can have precise information to help early and precise approximation of mental health disruptions, especially during crisis events. Nonetheless, these algorithmic applications need to be complemented with human follow-up to glean a holistic understanding of the individual’s needs. The automation provided by the ML models can help prioritize cases.

Our study also has implications for the use of private social media data in health research. Private social media could express the mental state of an individual more intimately; however, it is also likely to be more sensitive. Therefore, it is imperative to responsibly design data-sharing paradigms, especially since these data can be used to estimate an individual’s health. One possibility is to follow the paradigm in our study. First, the participants downloaded a full copy of their data. Then they submitted the data to us and our portal showed them a sample of tables we stored with deidentified information. They were able to download a full copy of the data we kept on the server as well. Through this approach, the participants were more mindful of the data provision process, and they had full transparency on what data are outside the social media platform itself. First responders and other clinical support can consider establishing similar mechanisms that ensure that enrollment is not only voluntary but also respects agency.

### Limitations

Our study was aimed at demonstrating feasibility and our data included a long span of data, albeit for a small number of participants. The small number could lead to overfitting of our ML models. Given this concern, we took rigorous steps to reduce problems of overfitting, such as the use of leave-one-participant-out cross-validation, and presented pooled accuracy results (as opposed to mean accuracy). Having said that, our data set has too few participants to confidently claim that our models would work well off-the-shelf with new individuals. Instead, this paper provides evidence for larger recruitments to build more stable and generalizable models for prediction of crisis-induced mental health disruptions.

In addition, this study was conducted retrospectively in reference to the actual crisis. Social media is only one source of archival data. Future studies may consider leveraging other sources of data and, thus, provide a more holistic perspective on well-being in times of crisis. For instance, on-campus routers have been retroactively repurposed to understand student behaviors related to performance and health [23,40,41]. Combining multiple archival sources of data from the university infrastructure can help with situational preparedness.

Finally, the disruption to mental well-being was self-reported, but future studies may be able to complement these labels with complementary signals (eg, absenteeism or routine disruptions). Through these methods, researchers can mitigate the recall bias associated with self-reported labels. In fact, future studies can extend our temporal approach to identify anomalies in trends to not only identify *whether* an individual is vulnerable to health disruption but also pinpoint exactly *when* the disruptions are likely to take place.

### Conclusions

The purpose of this research was to establish the feasibility of social media as a way to understand disruptions induced by a global crisis—COVID-19, among a particularly vulnerable population—college students. Our results indicate promise for larger and longer studies to understand the crisis-induced mental health disruptions. Given the retroactive nature of our approach, we stand to study a variety of crisis scenarios that may affect student populations.

## Abbreviations

CRISIS: Coronavirus Health and Impact Survey
LIWC: linguistic inquiry and word count
ML: machine learning

## Data Availability

The de-identified data used in our analyses will be made available by the authors on reasonable request. The data are not publicly available because of the sensitive nature of mental health and private aspects of the social media data.
